# Establishment of haemoglobin A_2_ reference intervals in Pretoria, South Africa: A retrospective secondary data analysis

**DOI:** 10.4102/ajlm.v11i1.1841

**Published:** 2022-08-12

**Authors:** Cailin Nieuwenhuizen, Tshiphiri Netshidzivhani, Johan Potgieter

**Affiliations:** 1 Department of Haematology, Faculty of Health Sciences, University of Pretoria, Pretoria, South Africa; 2 Department of Haematology, Tshwane Academic Division, National Health Laboratory Service, Pretoria, South Africa

**Keywords:** Haemoglobin A2, reference range, reference interval, beta thalassemia, high-performance liquid chromatography

## Abstract

**Background:**

Haemoglobinopathies are one of the most common inherited diseases worldwide. Quantification of haemoglobin A_2_ is necessary for the diagnosis of the beta thalassaemia trait. In this context, it is important to have a reliable reference interval for haemoglobin A_2_ and a local reference range for South Africa has not been established.

**Objective:**

This study aimed to establish reference intervals for haemoglobin A_2_ using stored patient laboratory data.

**Methods:**

This descriptive study used retrospective data to evaluate haemoglobin A_2_ levels determined using high-performance liquid chromatography at the National Health Laboratory Service haematology laboratory in Pretoria, South Africa. All tests performed from 01 October 2012 to 31 December 2020 were screened for inclusion; of these, 144 patients’ data met the selection criteria. The reference interval was calculated using descriptive statistics (mean and standard deviation) with a 95% confidence interval.

**Results:**

Analysed data from enrolled patients showed a normal distribution. The mean age of the patients was 40 years (range: 3–84 years). The reference interval for haemoglobin A_2_ calculated from this data was 2.3% – 3.6%. The minimum haemoglobin A_2_ was 2.3% and the maximum was 3.9% with a mean of 2.95% and a standard deviation of 0.357%.

**Conclusion:**

A normal reference interval has been established for the population served by the laboratory that will assist with accurate diagnosis of the beta thalassaemia trait. This reference interval may also be useful to other laboratories that employ the same technology, especially smaller laboratories where obtaining a sufficiently large number of normal controls may be challenging.

## Introduction

Reference intervals are an integral part of any functioning clinical laboratory. Reference intervals are often the endpoint used by clinicians in the management of patients and in clinical decision-making. It has been estimated that 80% of medical decisions are made from laboratory results.^1^ The value of the results produced by a laboratory is largely influenced by the quality of the reference interval used in the interpretation of that result.^2^

An example of where reference intervals are of diagnostic utility is in the diagnosis of the beta thalassaemia trait. The increase in haemoglobin A_2_ (HbA_2_) level is the most important laboratory parameter for the identification of carriers of beta thalassaemia, and is considered diagnostic in the appropriate clinical context.^3^ Accurate quantification of HbA_2_ in the haematology laboratory is therefore essential to allow for routine diagnosis of the beta thalassaemia trait.^4^ This highlights the value of reliable reference intervals.

Beta thalassaemia is inherited in an autosomal recessive pattern and the beta thalassaemia trait is estimated to have a prevalence of 1.5% worldwide, affecting approximately 80–90 million people.^5^ Africa has a considerable disease burden in terms of haemoglobinopathies and in particular beta thalassaemia with 1520 conceptions affected annually, Western Africa accounting for the majority of cases.^6^

Reference intervals are established through a validation process with a statistically adequate number, ideally 120, of healthy reference individuals.^7^ This needs to be done for all reagents and instrument combinations. However, even the Clinical and Laboratory Standards Institute guidelines recognise that this is not feasible for many laboratories and finding a cohort of 120 healthy individuals is not feasible for every test.^8^ Alternatively, reference intervals may be verified, with only 20 samples needed, or transferred provided that the analytic system and the test population are comparable.^8^ However, verification and transfer of reference intervals is not ideal and these methods have their own disadvantages.^8^

Every laboratory that performs HbA_2_ testing is responsible for establishing its own reference interval. This is done by quantifying the HbA_2_ percentage in a cohort of healthy adults who do not have iron deficiency or the thalassaemia trait.^3^ The reference interval is a range that should be calculated including individuals with characteristics that are comparable to the reference group so that the reference interval can be correctly applied to the population serviced by the laboratory.^7^ Given the importance of HbA_2_ reference ranges, considerable work has been done internationally on normal reference intervals for HbA_2_. However, there is a paucity of literature from Africa with no published reference intervals for HbA_2._

In keeping with good laboratory practice, a need was identified to determine HbA_2_ reference intervals in a local African population for the Department of Haematology of the National Health Laboratory Service (NHLS), Tshwane Academic Division (TAD), Pretoria, South Africa. Using a sufficient number of results from medical records would provide a healthy cohort for establishing a reference interval without the need to recruit healthy individuals. This is particularly useful for a test that is not routinely requested and only offered by specialised laboratories. In order to establish a reference interval for HbA_2_ without the limitations inherent to traditional methods of establishing reference intervals, we made use of previously reported normal high-performance liquid chromatography (HPLC) results from the NHLS Department of Haematology.

## Methods

### Ethical considerations

Approval was obtained from the Academic Affairs, Research and Quality Assurance Department of the NHLS. The study protocol was approved by the Faculty of Health Sciences Research Ethics Committee of the University of Pretoria (protocol number 518/2019). Patient consent was waived by the ethics committee as the study was conducted using historical data. Participants’ information was treated with confidentiality. Each participant was allocated a unique study number to ensure anonymity.

### Study design and setting

This was a descriptive study using retrospective data from blood test results stored in the Laboratory Information System of the NHLS at the Department of Haematology, TAD, and Vermaak and Partners Path Care Pathology group.

### Study population and sampling strategy

**FIGURE 1:**
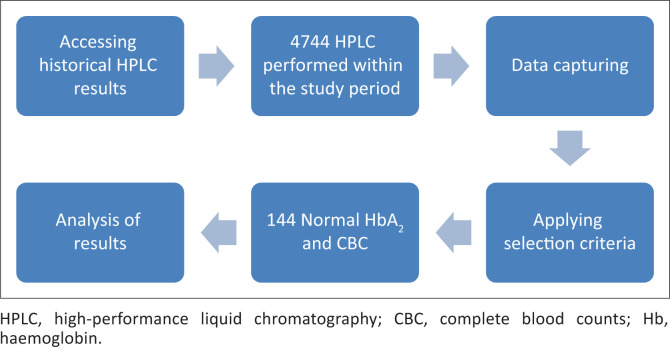
Flow diagram of the study design, Pretoria, South Africa, 01 October 2012 – 31 December 2020.

The study population comprised patients investigated by TAD, NHLS, and included patients from both high-income and low-income settings and represented a variety of ethnicities. All HPLC results of tests performed at the NHLS TAD from 01 October 2012 to 31 December 2020 were screened for inclusion (Figure 1). The following selection criteria were applied: HPLC performed within the study period and normal HPLC results of patients who also had a corresponding complete blood counts (CBC) were included in this study. Results of individual patients were excluded if they were aged two years or younger, were anaemic (haemoglobin < 12.5 g/dL), had a mean cell volume < 75 fL or > 100 fL, had a mean corpuscular haemoglobin below 27 pg, had a red cell distribution width of > 15% or had variant haemoglobin, inclusive of haemoglobin S, detected in their haemoglobin electrophoretic result. Participants that did not meet the selection criteria or met the exclusion criteria were excluded from the study and subsequent analysis. A minimum sample size of 120 patient is required in order to establish a reference interval.^8^

### Data collection

Apart from HPLC reports, corresponding results of CBC, thyroid stimulating hormone, serum folate, serum vitamin B12 and ferritin level, performed within a week of the taking of the HPLC specimen, were evaluated. Only normal HPLC results were included in the analysis.

The HbA_2_ levels were determined in the haematology laboratory using the HPLC D10 instrument (Bio-Rad® Laboratories, Hercules, California, United States). The D10 instrument uses ion-exchange HPLC technology to analyse haemoglobin. All analyses were performed according to good laboratory practice and the manufacturer’s recommendations. The haematology laboratory at the NHLS TAD has been accredited by the South African National Accreditation System. Appropriate controls and calibrators were used throughout the study. The Lyphochek® Hemoglobin A_2_ Control Level 1 and 2 (Bio-Rad Laboratories, Irvine, California, United States) were used as controls and the D10^TM^ Dual Program HbA_2_/F/A_1C_ Calibrator/Diluent Set (Bio-Rad^®^ Laboratories, Hercules, California, United States) was used for calibration.

The blood test results listed above were captured in a Microsoft Excel (Microsoft Corporation, Redmond, Washington, United States) spreadsheet. Exclusion criteria were applied after which 144 samples with normal HbA_2_ levels were identified, and these were used for calculation of reference intervals.

### Data analysis

The descriptive statistics mean, median, standard deviation and inter-quartile range, with 95% confidence interval, and the 2.5th and 97.5th percentiles were used to describe the continuous variables such as HbA_2_ levels. The two-sample *t*-test, or non-parametric alternative were used to compare group means. Pearson’s correlation was used to measure correlations between HbA_2_ levels and age, as well as other continuous variables such as CBC parameters. Tests were evaluated at 5% level of significance. All analyses were done using Stata 15 (StataCorp, College Station, Texas, United States) software.

## Results

**FIGURE 2:**
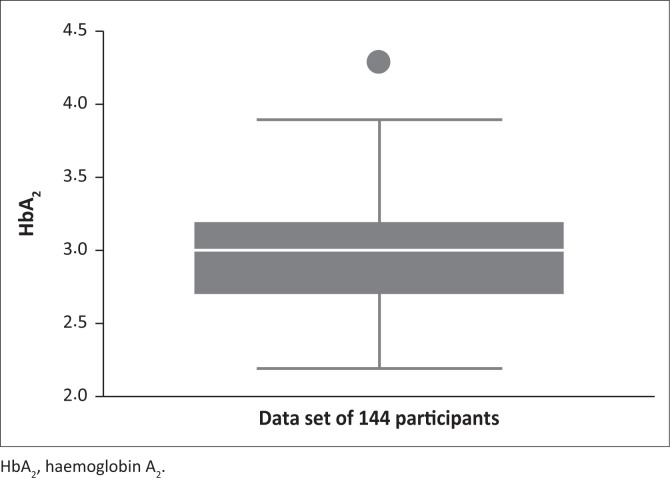
Box-plot of haemoglobin A_2_ distribution, Pretoria, South Africa, 01 October 2012 – 31 December 2020.

The mean age of the 144 patients included in this data analysis was 40 years (range 3–84 years). The study population comprised 67 female and 77 male patients. After a single outlier of 4.3% was excluded from the analysis, the mean HbA_2_ value was 2.95% with the range between 2.2% and 3.9%. The standard deviation was 0.357% (Figure 2).

**FIGURE 3:**
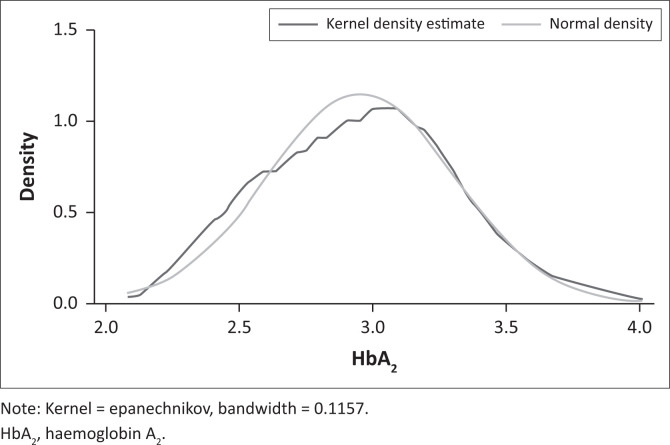
Kernel density estimate demonstrating the normal distribution of data, Pretoria, South Africa, 01 October 2012 – 31 December 2020.

Data were normally distributed as indicated in the Kernel density estimation (Figure 3). The HbA_2_ reference interval established from this data set was 2.3% – 3.6%.

**FIGURE 4:**
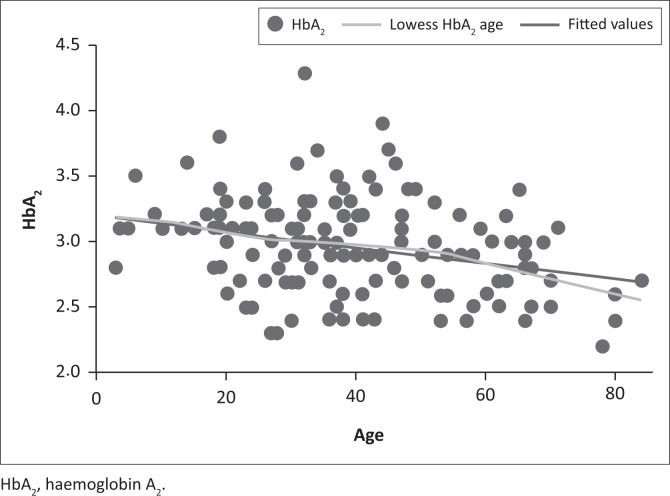
Scatterplot of haemoglobin A_2_ and age distribution, Pretoria, South Africa, 01 October 2012 – 31 December 2020.

A sex comparison was performed by *t*-test to compare the mean HbA_2_ of male patients to that of female patients in an attempt to identify possible bias. No significant difference was found (*p* = 0.1328). The correlation between age and HbA_2_ was also assessed. A trend towards lower HbA_2_ values with increased age was appreciated (Figure 4), with a Pearson correlation coefficient of –0.2826.

## Discussion

The HbA_2_ reference interval established from this data set was 2.3% – 3.6%. Normal reference intervals for HbA_2_ have been published for other patient populations. A study performed at the Leiden University in the Netherlands, using the Variant Classic HPLC (Bio-Rad^®^) platform, reported a HbA_2_ reference interval of 2.3% – 3.5%.^3^ Despite the difference in study population, the reference interval determined in this current study is comparable to that of the Leiden group which used a similar method, that is, HPLC (Bio-Rad^®^) technology. Han et al. reported a reference interval of 2.3% – 3.1% for HbA_2_ in a Chinese population of reproductive age.^9^ However, these investigators used a CAPILLARYS2 instrument (Sebia, France) to generate their data.^9^

Most evidence suggests that HbA_2_ of > 4% is indicative of beta thalassaemia trait with almost 100% sensitivity and 90% specificity.^10^ A grey zone between 3.1 and 3.9 is generally accepted as reported in a comprehensive review.^11^ This often poses a diagnostic challenge. Studies have been conducted to identify the presence of mutations in these individuals.^10,11,12,13,14^ Giambona et al. found that 80% of patients in this group in an Italian population were negative for molecular defects, and the most significant finding was the presence of beta thalassaemia gene mutations found mostly in patients with HbA_2_ in the region of 3.5–3.9 and mean cell volume < 80 fL.^11^

The upper limit of the normal reference interval established in this current study does fall within the previously described ‘grey zone’. However, in the presence of a normal CBC, the possibility of an underlying carrier state in these patients remains small. This highlights the importance of interpreting HbA_2_ within the clinical context, taking into consideration the CBC parameters and iron studies.

There appears to be a weak association between a decreasing HbA_2_ value and increasing age; this was true even when looking at subsets of age and when excluding patients aged 70 years or older. Although this trend was seen, when calculating the reference interval by age, the reference interval remained 2.3–3.6 when rounded to one decimal place. Therefore, this was not a significant finding.

In this current study we used data available on the laboratory information system in order to establish a reference interval. This represents a novel approach in our setting. Data mining is emerging as an alternative to the traditional direct a priori method. Data mining makes use of electronic data records and statistical techniques to determine the healthy population within a data set in order to establish a reference interval.^15^ The electronic data records may be obtained from insurance claims, electronic health records as well as a variety of other sources.^16^ All sources require data capturing platforms which allow for database management in order to deal with the enormous volume of information currently being generated as well as the complexity of analysing and interpreting the data.

The analytical methods that have been employed to establish reference intervals include The Hoffmann method, Bhatacharya method and more recently the Truncated Maximum Likelihood method.^15^ The Truncated Maximum Likelihood method employs complex statistical algorithms that make use of maximum likelihood estimation and require 4000 data points in order to establish a robust reference interval.^15^ Currently there is still hesitancy regarding the use of indirect methods but it is likely to be used to establish many reference intervals in the future. Indirect methods may be particularly useful for tests that are not routinely performed as screening tests in the healthy population.^17^ Data mining has many advantages: it is less costly as the blood results of large cohorts of individuals are readily available, it is faster, and it can even be considered more ethical.^15^ A study conducted by Katayev et al. showed that reference intervals could be reliably and reproducibly established using data mining. Reference intervals were calculated for eight analytes and were found to be comparable to already accepted published reference intervals.^17^ Although our study was small and the data were captured manually, it does highlight the potential of using laboratory information records to glean valuable data with relatively low cost and fewer limitations than are inherent to establishing reference intervals in the conventional manner by direct population sampling.

### Limitations

One of the limitations of this study was the inability to exclude all confounding factors. Although our study could be improved on by only including patients who have been tested for all confounding factors for HbA_2_, this would only be feasible if a large number of data sets were included. Another option would be to use a larger cohort and an algorithm that does not require the exclusion of all confounders. A normal CBC was used as a surrogate marker of a nutritional deficiency, and this remains a limitation. It should be noted that all published confounders are not routinely considered when establishing reference intervals for HbA_2_ or when interpreting HPLC results.

### Conclusion

A normal HbA_2_ reference interval of 2.3% – 3.6% has been established for the population served by the laboratory. This will assist with the interpretation of results. The reference interval could also be useful to other laboratories, especially smaller laboratories where obtaining a sufficiently large number of normal controls may be challenging. The inability to exclude all of the confounding factors that influence HbA_2_ levels needs further research.
